# Effects of an Integrated Breast Self-Examination Education Program Incorporating Practice with a Wearable Breast Examination Simulator on Breast Cancer Knowledge and Health Beliefs: A Randomized Controlled Trial

**DOI:** 10.3390/healthcare14193191

**Published:** 2026-09-28

**Authors:** Aysun Badem, Emine Derya İster, Merve Gülpak

**Affiliations:** 1 Department of Obstetrics and Gynecology Nursing, Faculty of Health Sciences, Kahramanmaraş Sütçü İmam University, 46100 Kahramanmaraş, Türkiye; 2 Department of Internal Medicine Nursing, Faculty of Health Sciences, Kahramanmaraş Sütçü İmam University, 46100 Kahramanmaraş, Türkiye

**Keywords:** breast cancer, breast self-examination, breast awareness, health beliefs, wearable simulator, health education, randomized controlled trial

## Abstract

Background: Recognizing breast changes and seeking timely medical evaluation are components of breast awareness. This study evaluated the effects of an integrated breast self-examination (BSE) educational intervention incorporating wearable simulator practice on breast cancer knowledge and health beliefs. Methods: This two-arm, parallel-group randomized controlled trial included 95 women aged 40 years or older (intervention, *n* = 49; control, *n* = 46). The intervention group received an integrated intervention comprising individualized face-to-face education, simulator-supported hands-on practice, printed and electronic educational materials, and monthly reminders over three months. The control group received no intervention. Breast cancer knowledge and health beliefs were assessed at baseline and after three months using the Comprehensive Breast Cancer Knowledge Test and the Champion Health Belief Model Scale. Results: At three months, after adjustment for baseline scores, the intervention group had higher general knowledge and curability scores than the control group (*p* < 0.001). For total knowledge, the sensitivity analysis without the interaction favored the intervention group (adjusted mean difference = 3.14, 95% CI: 2.14–4.15; *p* < 0.001); the interaction analysis was exploratory (*p* = 0.041). Adjusted perceived benefit, confidence, and health motivation scores were also higher in the intervention group (*p* < 0.001). Conclusions: An integrated educational intervention incorporating practice with a wearable breast simulator was associated with higher follow-up scores for breast cancer knowledge, perceived benefits, confidence, and health motivation than no intervention (ClinicalTrials.gov: NCT06886594).

## 1. Introduction

Breast cancer is the leading cause of cancer-related mortality and the most prevalent form of cancer among the global female population [1]. According to statistics reported by the World Health Organization (WHO), cancer is the second leading cause of mortality worldwide. In 2020, the global mortality rate due to cancer was 10 million deaths. According to reports, breast cancer is the most prevalent type of cancer among women. When all cancers are considered, breast cancer ranks fifth in terms of mortality [2]. In the context of cancerous diseases affecting Turkish women, breast cancer has been identified as the most prevalent type, accounting for approximately 25% of all diagnosed cases [3]. The following factors have been identified as risk factors for breast cancer: advanced age, a history of cancer in a first-degree relative, environmental factors, lifestyle habits, hormonal factors, and late childbearing, typically after the age of 35 [4]. The incidence and mortality of breast cancer may be influenced by socioeconomic status. A comparison of high-income and low-income countries reveals that while breast cancer incidence is higher in the former, mortality rates are lower in the latter [5]. The disparities in breast cancer mortality rates among nations are associated with various factors. These include the improved management of risk factors in high-income countries, enhanced access to educational resources, and increased adherence to screening and surveillance programs that facilitate early detection of the disease. High-income countries have been shown to generally have better access to primary health care and more public cancer centers offering improved diagnostic and treatment options. In order to address the significant health burden posed by breast cancer, future efforts must include the following: first, the implementation of screening procedures for risk factors; second, the tracking of global data relevant to the disease; third, the promotion of early detection; fourth, the provision of health guidance; and fifth, the implementation of improved interventions and treatments to reduce the risk of the disease [1,6,7]. Early diagnosis of breast cancer is paramount in reducing mortality and ensuring a high quality of life for patients [8].

In Türkiye, the Ministry of Health has issued guidelines recommending a series of measures as part of a comprehensive breast cancer screening program. These measures include monthly counseling on breast self-examination (BSE), annual clinical breast examinations, and biennial mammography for women aged 40 to 69 years [9]. BSE is a simple procedure performed monthly at the individual level. The purpose of BSE is to examine and detect abnormalities in the breast [10]. A comprehensive review of the literature indicates that appropriate and regular BSE may help women recognize breast changes promptly and improve breast cancer awareness [11]. BSE is an accessible and non-invasive practice that women can perform privately without requiring additional equipment or a healthcare visit [12]. Regular BSE practice remains limited, and studies conducted in different populations have identified insufficient knowledge and unfavorable attitudes toward BSE among women [13,14,15,16]. According to Kalliguddi et al., the participants’ knowledge of BSE was moderate, with only 0.5% demonstrating a high level of knowledge [17]. A multitude of studies have identified a series of factors that have the potential to influence the probability of developing breast cancer. These factors encompass beliefs and attitudes concerning the disease, awareness of risk factors, social influence, and levels of self-efficacy [18,19,20,21]. Women may postpone clinical examinations due to fear of developing cancer and a lack of knowledge, which may result in delayed diagnosis [22]. Increasing women’s awareness of the importance of BSE, integrating BSE education into health curricula, and encouraging regular BSE practice may help women recognize breast changes and seek timely medical evaluation [23].

Since 2008, the National Cancer Screening Program, administered by KETEM, has been implemented in Türkiye. The objective of an effective screening method is to reach 70% of the population [24]. The current coverage of breast cancer screening in Türkiye is 30–35%, which falls significantly below the established target [25]. The finding that screening programs are falling short of the desired level suggests that BSE is a significant concern for women. Women aged 40 years and older were selected to align the educational intervention with the target population of the national breast cancer screening program [25], rather than to suggest that breast awareness or BSE education is relevant only to this age group. The present study examined whether an integrated BSE educational intervention incorporating wearable simulator practice influenced breast cancer knowledge and health beliefs among women aged 40 years and older. While the benefits of BSE are widely recognized, randomized controlled trials evaluating integrated, practice-based educational interventions incorporating wearable breast simulators remain limited. Previous studies have predominantly emphasized theoretical or video-based education, which may fall short in enhancing women’s practical skills and confidence in performing BSE. This study addresses this gap by evaluating an integrated intervention combining individualized face-to-face education, hands-on practice with a wearable simulator, printed and electronic educational materials, and follow-up reminders.

## 2. Methods

### 2.1. Aim and Hypothesis

The objective of this study was to evaluate the effects of an integrated BSE educational intervention incorporating wearable simulator practice on breast cancer knowledge and health beliefs among women aged 40 years and older.

The hypotheses of this study were as follows:

**H1.**  *An integrated BSE educational intervention incorporating wearable simulator practice increases breast cancer knowledge among women aged 40 years and older*.

**H2.**  *An integrated BSE educational intervention incorporating wearable simulator practice positively affects breast cancer-related health beliefs among women aged 40 years and older*.

#### Design

Women were randomly assigned to one of two parallel groups, and outcome assessment was conducted by a blinded assessor. Participants and the researcher who delivered the training could not be blinded because the intervention involved hands-on simulator-based education. Follow-up assessments were therefore conducted by a trained assessor who was unaware of the participants’ group assignments and had no role in delivering the intervention. An independent statistician analyzed the data.

### 2.2. Location and Characteristics of the Research

The study was conducted from June to December of 2024, and it was conducted at the gynecology outpatient clinic of Kahramanmaraş Sütçü İmam University Health Practice and Research Hospital. Eligible women were randomly assigned to either the intervention or control group. The allocation of subjects into these groups was determined by a simple randomization technique.

### 2.3. Sample Size Calculation

The sample size was calculated based on the post-intervention perceived barriers subscale scores of the Champion Health Belief Model Scale reported in a previous study conducted in Türkiye (intervention group: 23.97 ± 2.40; control group: 25.72 ± 3.51) [26]. Based on these values, the standardized effect size was calculated as Cohen’s d = 0.58. Using a two-sided independent-samples *t*-test, an α level of 0.05, a statistical power of 80%, and a 1:1 allocation ratio, the minimum required sample size was determined as 96 participants (48 per group). To account for potential loss to follow-up, 100 women were recruited and randomized, with 50 participants allocated to each group.

### 2.4. Eligibility Criteria

Women were eligible to participate if they were aged 40 years or older, had completed at least primary education, were able to communicate effectively, and voluntarily agreed to participate in the study. Women were excluded if they had a history of breast cancer, had previously received training on breast cancer or breast self-examination from a healthcare professional, regularly performed breast self-examination, worked as healthcare professionals, or had completed an educational program in a health-related field.

### 2.5. Participants

A total of 184 women were assessed for eligibility. Of these, 82 did not meet the inclusion criteria and two declined to participate. The remaining 100 women were randomized in a 1:1 ratio to the intervention group (*n* = 50) or the control group (*n* = 50). During follow-up, one participant in the intervention group and four participants in the control group could not be reached by telephone; therefore, their three-month follow-up data could not be obtained. The final analysis included 95 participants (intervention group, *n* = 49; control group, *n* = 46) (Figure 1).

### 2.6. Randomization

Once eligibility had been established and written informed consent was provided, participants received individual study identification numbers. A computer-generated random allocation sequence was prepared before participant recruitment using Research Randomizer, with a 1:1 allocation ratio and simple randomization. The sequence was generated by a researcher who was not involved in delivering the training and was maintained in a concealed allocation list. Participants were assigned sequentially to the intervention or control group according to the next available allocation. The researcher responsible for participant enrolment had no access to the allocation list and was unaware of the upcoming group assignment. Women who did not meet the eligibility criteria or declined to participate were not assigned a study number and were not entered into the randomization sequence.

### 2.7. Blinding

Due to the nature of the educational intervention, participants and the researchers who delivered the training could not be blinded to group allocation. However, baseline and three-month follow-up data were collected by an experienced independent interviewer who was not involved in delivering or monitoring the intervention and had no access to the randomization list or allocation information.

### 2.8. Data Collection

We collected data using the Personal Information Form, the Champion Health Belief Model Scale (CHBMS), and the Comprehensive Breast Cancer Knowledge Test (BCKT).

### 2.9. Personal Information Form

The Personal Information Form is composed of a series of inquiries concerning women’s sociodemographic characteristics and their family history of breast cancer. The form encompasses questions on subjects’ age, marital status, educational attainment, family history of breast cancer, number of births, breastfeeding status, and duration of breastfeeding [26]. The information form was developed for the purposes of this study and has not been previously published.

### 2.10. Comprehensive Breast Cancer Knowledge Test (BCKT)

The Comprehensive Breast Cancer Knowledge Test was developed by Stager (1993) in 1993 [27]. The Turkish validity and reliability study of the scale was conducted by Başak (2015) [28]. The scale comprises a total of 20 items, which are to be answered using a “true–false” response format. The scale contains 8 true and 12 false statements. The BCKT incorporates two dimensions: general knowledge and curability. Of the 20 items, 12 assess general breast cancer knowledge and risk factors, while the remaining eight focus on breast cancer treatability. The scale demonstrates adequate internal consistency, with a Cronbach’s alpha coefficient of 0.71 [28]. In this study, the Cronbach’s alpha coefficient of the BCKT was 0.78.

### 2.11. Champion Health Belief Model Scale (CHBMS)

The Health Belief Model, developed by Champion in 1984, consists of six dimensions, including six concepts and 43 items. The sub-dimension of perceived susceptibility to breast cancer (3 items) refers to the perceived personal risks of developing breast cancer. The construct of perceived seriousness, comprising seven items, is defined as the extent to which the individual perceives the threat of breast cancer. The construct of perceived benefit, encompassing five items, is defined as the perceived advantage of BSE. The perceived barriers subscale (11 items) refers to perceived barriers related to BSE, while the perceived confidence subscale (10 items) refers to perceived individual competence to perform BSE to identify abnormal breast masses. The health motivation sub-dimension (7 items) expresses the individual’s interests and concerns about their health status. The internal consistency Cronbach alpha coefficient of the scale, the reliability and validity of which were studied by Champion, varies between 0.69 and 0.90 for the sub-dimensions [29]. Karayurt and Dramalı’s (2007) adaptation of the scale into Turkish yielded Cronbach alpha reliability coefficients for the sub-dimensions ranging from 0.58 to 0.89 [30]. In the present study, the Cronbach alpha reliability coefficients of the sub-dimensions of the scale were found to range from 0.73 to 0.94.

### 2.12. Study Procedures

#### Intervention Group

Before the intervention, baseline data were collected by the blinded independent interviewer using the Personal Information Form, the Champion Health Belief Model Scale (CHBMS), and the Comprehensive Breast Cancer Knowledge Test (BCKT).

Each participant received individual, face-to-face theoretical and practical training in a single session lasting approximately 30 min. The training was conducted in an unoccupied examination room in the gynecology outpatient clinic to ensure privacy and minimize interruptions. It was delivered by the first researcher, who held a doctoral degree in Obstetrics and Gynecology Nursing. The training content was developed in accordance with the Turkish Ministry of Health Breast Cancer Prevention, Screening, Diagnosis, Treatment, and Follow-up Clinical Guidelines 2020 (Version 1.0) [31]. Participants were given an opportunity to ask questions during and after the training.

Practical BSE training was conducted using a wearable silicone breast examination simulator (model L50; Figure 2). The simulator contained two benign masses, four malignant masses, a palpable left axillary abnormality, left nipple retraction, and a peau d’orange-like appearance of the left breast. The first researcher demonstrated BSE according to the steps described in the brochure published by the Cancer Early Diagnosis, Screening and Training Center of the Turkish Ministry of Health. Each participant then wore the simulator and practiced BSE under the supervision of the second and third researchers. A formal structured skills checklist was not used; the researchers assessed participants’ completion of the BSE steps through direct observation, using the sequence described in the Ministry of Health brochure as a guide. Practice continued until the participant correctly completed all BSE steps and identified at least three of the six palpable masses. Identification of any three masses was accepted as a pragmatic minimum practice benchmark intended to balance demonstration of the palpation technique with the feasibility of the approximately 30-min individualized session; it was not used as a validated measure of BSE competence. Reaching this threshold did not immediately terminate the practice, as participants completed the full BSE procedure and were encouraged to continue examining the simulator with guidance and immediate feedback.

Immediately after the training, each participant was given a printed copy of the BSE brochure. The educational video and an electronic copy of the brochure were also shared via WhatsApp. Reminder messages encouraging monthly BSE practice were sent via WhatsApp at one, two, and three months after the training. Questions raised by participants during the follow-up period were answered by the researchers. At the three-month follow-up, the CHBMS and BCKT were readministered by the same blinded independent interviewer.

### 2.13. Control Group

Baseline data were collected from participants in the control group by the same blinded independent interviewer using the Personal Information Form, CHBMS, and BCKT. Participants received no breast cancer or BSE education and no reminders to perform BSE during the three-month follow-up period. To maintain contact and minimize loss to follow-up, the researchers sent a brief neutral WhatsApp message once a month asking about the participants’ general well-being and confirming their continued participation in the study. These messages did not include educational content or recommendations regarding BSE. At the end of the three-month follow-up period, the CHBMS and BCKT were readministered by the same blinded independent interviewer. After completion of the post-test assessment, participants received the BSE educational video and electronic brochure via WhatsApp.

### 2.14. Human Participant Compliance Statement

This study was reviewed and approved by the Medical Research Ethics Committee of Kahramanmaraş Sütçü İmam University (Session No: 2023/25, Decision No: 14, dated 8 December 2023). Institutional permission was obtained from the Kahramanmaraş Sütçü İmam University Health Practice and Research Hospital. All procedures involving human participants were conducted in accordance with the ethical standards of the institutional and/or national research committee and with the 1964 Helsinki Declaration and its later amendments. Informed consent was obtained from all women who participated in the study. The trial was retrospectively registered at ClinicalTrials.gov on 20 March 2025 (NCT06886594). This was due to a lack of awareness of the prospective registration requirement at the time of participant enrollment.

### 2.15. Data Analysis

The data obtained in the study were analyzed using SPSS (Statistical Package for the Social Sciences) for Windows, version 22.0. Descriptive statistics, including the number, percentage, mean, and standard deviation, were used to evaluate the data. Chi-square analysis was applied to test the homogeneity of the intervention and control groups according to demographic variables. The suitability of the data for normal distribution is contingent upon the skewness and kurtosis values falling within the range of ±3, as delineated by the skewness and kurtosis coefficients [32]. In this study, the skewness coefficient of the data ranged from 0.039 to 1.296, and the kurtosis coefficients ranged from 0.037 to 1.034. These findings indicate that the data were normally distributed. Changes from pre-test to post-test within each group were analyzed using paired-samples *t*-tests. The intervention and control groups were compared at both pre-test and post-test using independent-samples *t*-tests. These *t*-tests were considered supplementary analyses of unadjusted score changes, and their full results are presented in Supplementary Tables S1 and S2. ANCOVA was used as the primary analysis of intervention effects, with post-test scores as the dependent variables and the corresponding baseline scores as covariates. For the BCKT total score, a sensitivity analysis was conducted using a simpler baseline-adjusted model without the group × baseline interaction. Because the interaction analysis was not prespecified, the interaction and the corresponding conditional comparisons were treated as exploratory. The assumptions of ANCOVA, including linearity, homogeneity of regression slopes, normality of residuals, and homogeneity of error variances, were assessed. For the six CHBMS subscale comparisons, a Bonferroni-adjusted significance threshold of *p* < 0.008 was applied. A two-sided *p* < 0.05 was considered statistically significant, except for the six CHBMS subscale comparisons, for which *p* < 0.008 was used. Analyses were conducted as complete-case analyses and included the 95 participants (intervention, *n* = 49; control, *n* = 46) who completed the three-month follow-up. The five randomized participants with missing follow-up outcome data were excluded from the analyses, and missing data were not imputed; therefore, the analyses did not constitute a strict intention-to-treat approach.

## 3. Results

The sociodemographic and obstetric characteristics of the intervention and control groups are presented in Table 1. No statistically significant between-group differences were observed in the sociodemographic or obstetric characteristics, indicating that the groups were comparable at baseline (all *p* > 0.05). The mean age was 46.92 ± 6.39 years in the intervention group and 48.59 ± 7.09 years in the control group. Most participants in both groups were married, had no family history of breast cancer, and had previous breastfeeding experience.

Unadjusted pre-test and post-test BCKT means and standard deviations for the intervention and control groups are presented descriptively in Table 2. The full results of the supplementary independent- and paired-samples *t*-tests are provided in Supplementary Table S1.

After adjustment for baseline scores, post-test General Knowledge and Curability scores were significantly higher in the intervention group than in the control group (adjusted mean differences = 1.81 and 1.37, respectively; both *p* < 0.001). In the sensitivity analysis using a simpler baseline-adjusted model without the group × baseline interaction, the adjusted post-test BCKT total score was higher in the intervention group than in the control group (adjusted means = 13.23 and 10.08, respectively; adjusted mean difference = 3.14, 95% CI: 2.14–4.15; F(1, 92) = 38.44, *p* < 0.001, partial η^2^ = 0.295). An exploratory group × baseline interaction was also statistically significant (F(1, 91) = 4.29, *p* = 0.041, partial η^2^ = 0.045). Conditional comparisons from the exploratory model showed higher adjusted total scores in the intervention group at low, mean, and high baseline levels (adjusted mean differences = 2.08, 3.13, and 4.18, respectively; all *p* ≤ 0.004). This exploratory pattern was characterized by decreasing modeled follow-up estimates in the control group as baseline knowledge increased, whereas the intervention-group estimates changed little; therefore, it should be interpreted cautiously (Table 3).

For General Knowledge and Curability, the corresponding baseline score was included as the covariate. For the BCKT total score, the sensitivity analysis was conducted using a simpler baseline-adjusted model without the group × baseline interaction. The group × baseline interaction analysis was not prespecified and was therefore treated as exploratory. Because the exploratory interaction was statistically significant [F(1, 91) = 4.29, *p* = 0.041, partial η^2^ = 0.045], conditional group comparisons were performed at the mean baseline total score and at one standard deviation below and above the mean. CI, confidence interval; BCKT, Breast Cancer Knowledge Test; SD, standard deviation.

Unadjusted pre-test and post-test CHBMS subscale means and standard deviations for the intervention and control groups are presented descriptively in Table 4. The full results of the supplementary independent- and paired-samples *t*-tests are provided in Supplementary Table S2.

After adjustment for baseline scores, the intervention group had significantly higher post-test scores for perceived benefits, confidence, and health motivation than the control group (all *p* < 0.001). These differences remained significant at the Bonferroni-adjusted threshold of *p* < 0.008. No significant between-group differences were found for susceptibility, seriousness, or perceived barriers (all *p* > 0.05) (Table 5). Overall, H1 was supported, whereas H2 was partially supported.

No intervention-related adverse events, including participant-reported discomfort or anxiety, were reported during the study.

## 4. Discussion

According to GLOBOCAN 2022, breast cancer accounted for an estimated 2.3 million new cases and 11.6% of all cancer cases worldwide [33]. In Türkiye, participation in breast cancer screening remains approximately 30% among the target population, and only 48.2% of breast cancer cases are diagnosed at the localized stage [25]. Improving women’s understanding of breast cancer and BSE may encourage greater engagement in early detection practices.

This study examined the effects of an integrated BSE educational intervention incorporating wearable simulator practice on breast cancer knowledge and health beliefs among women aged 40 years and older. After adjustment for baseline scores, the intervention group had higher post-test BCKT General Knowledge and Curability scores than the control group. For the BCKT total score, the sensitivity analysis without the interaction showed a higher adjusted post-test score in the intervention group than in the control group. The non-prespecified group × baseline interaction was treated as exploratory; although conditional comparisons favored the intervention group at low, mean, and high baseline levels, modeled control-group scores decreased as baseline knowledge increased. This unusual pattern warrants cautious interpretation, and whether the intervention effect varies according to baseline knowledge remains uncertain. Similarly, Karimian et al. (2022) reported improvements in women’s breast cancer- and BSE-related knowledge, attitudes, and practical skills following both video-based multimedia and face-to-face education [34]. In-person training may enhance adherence to recommended practices and help correct existing misconceptions concerning BSE [34]. A study conducted among female university students using a pretest–posttest design showed that educational intervention improved university students’ BSE-related knowledge, attitudes, and practices [35]. In a study conducted by Potluri et al., the objective was to increase the awareness of women residing in rural areas about breast cancer. Training increased women’s breast cancer knowledge and awareness [36]. Incesu et al. compared web-based and conventional BSE education among nursing students in a randomized controlled trial. Web-based BSE education improved students’ BSE skills but did not increase their BSE knowledge [37]. This discrepancy may be attributable to the characteristics of the sample group or the research methodology employed. In a related study, a breast simulator similar to the one used in the current study was utilized, and it was found that the knowledge levels of women who received BSE training increased [38]. The findings of this study, in concordance with the majority of the existing literature, showed that education delivered through different teaching strategies, including face-to-face, video-based, and simulator-supported methods, may increase women’s knowledge of breast cancer and BSE [34,35,36,38,39].

After adjustment for baseline scores, the intervention group had higher post-test perceived benefits, confidence, and health motivation scores than the control group. These differences remained significant after the Bonferroni adjustment. The descriptive pattern underlying these between-group differences showed little change in perceived benefits and only a modest increase in health motivation in the intervention group, alongside declines in both outcomes in the control group; therefore, these findings should not be interpreted as substantial absolute improvements within the intervention group. In the study by Rezaeian et al., perceived benefit scores were higher in the intervention group than in the control group following breast cancer screening education for women aged 40 years and older [39]. A study conducted by Elsawy et al. with visually impaired women revealed a statistically significant difference in total knowledge and total CHBMS scores before and after the BSE training program [40]. In another study involving 144 women in Iran, post-test mean scores for perceived susceptibility, seriousness, barriers, and benefits did not differ significantly between the intervention and control groups [41]. In the present study, no significant adjusted between-group differences were found in perceived susceptibility, perceived seriousness, or perceived barriers. However, significant intervention effects were observed for perceived benefits, confidence, and health motivation.

Within the intervention group, perceived susceptibility, seriousness, barriers, and confidence scores increased from pre-test to post-test. These within-group changes should not be interpreted as intervention effects independent of the between-group analyses. In particular, the increase in perceived barriers does not indicate a favorable outcome. After adjustment for baseline differences, there was no significant difference between the groups in perceived barriers. Nahidi et al. reported a significant improvement between the pre-test and post-test mean scores in perceived susceptibility, perceived seriousness, and perceived barriers sub-dimensions, favoring the post-test [41]. In the study by Pirzadeh et al. (2021), perceived susceptibility and seriousness did not change after training, whereas perceived benefits increased significantly [42]. Susceptibility and seriousness may be less responsive to a brief educational intervention because they involve personal risk perceptions and beliefs about the consequences of disease. The absence of significant between-group effects in these dimensions may also be related to the three-month follow-up period or the participants’ pre-existing beliefs.

In the present study, each woman in the intervention group received individualized education on breast cancer, as well as a brochure designed to provide comprehensive information on the subject. Following the theoretical training, BSE was practiced using a breast examination simulator. During the supervised practice session, participants were required to complete the BSE steps and identify at least three of the six palpable masses. This criterion served as a pragmatic minimum practice benchmark and was not intended as a validated measure of BSE competence. Together, theoretical education, simulator-based practice, immediate feedback, educational materials, and follow-up reminders constituted an integrated educational intervention. These complementary components may collectively explain the improvement in breast cancer knowledge and the higher adjusted follow-up scores observed for selected health beliefs.

Following the integrated educational intervention, breast cancer knowledge improved, and adjusted follow-up scores for perceived benefits, confidence, and health motivation were higher in the intervention group than in the control group. This integrated educational approach could therefore be considered a complementary component of breast awareness education in primary care and community settings. The wearable simulator is reusable and allows women to practice in a private setting. However, the present study did not evaluate objective BSE performance at follow-up, adherence to BSE over time, cost-effectiveness, implementation feasibility, participation in mammographic screening, or actual early diagnosis. Therefore, the findings do not establish that the intervention improves screening participation or clinical outcomes. Because the follow-up assessment was conducted at the end of the three-month reminder period, the observed differences should be interpreted as short-term effects and do not establish whether these effects persisted after the reminders ceased. Future studies could examine the longer-term sustainability of the observed effects, objective BSE skills, screening behaviors, and the feasibility and cost-effectiveness of delivering the intervention on a larger scale. In addition, average-risk participants enrolled in similar educational studies should be informed that the Canadian Task Force on Preventive Health Care has recommended that women not be advised to routinely perform formal BSE as a screening practice [43].

Although retrospective registration limits confirmation of outcome and analysis prespecification and may increase concern about selective reporting, the BCKT and CHBMS outcomes and measurement procedures were defined in the ethics-approved study protocol before recruitment; all BCKT and CHBMS outcomes collected at both assessments are reported, and the analyses were conducted by an independent statistician.

## 5. Conclusions

The integrated educational intervention incorporating simulator-supported hands-on BSE practice improved breast cancer knowledge and was associated with higher adjusted follow-up scores for perceived benefits, confidence, and health motivation among women aged 40 years and older. No significant effects were observed on perceived susceptibility, seriousness, or barriers. This educational approach may be used as a complementary component of breast awareness education in primary care and community health services.

## 6. Limitations

This study has several limitations. It was conducted at a single center among women attending an outpatient clinic, which may limit the generalizability of the findings. In addition, restricting eligibility to women aged 40 years or older and excluding women who had previously received professional breast cancer or BSE education or who regularly practiced BSE may limit the generalizability of the findings to younger women and to women encountered in routine screening or educational programs. Because the control group received no intervention, the additional individual attention and regular contact with the research team received by the intervention group, including monthly reminders, may have introduced an attention effect and contributed to the observed between-group differences independently of the educational content. The use of self-reported measures may have introduced response bias. The study focused on knowledge and health beliefs; objective BSE performance at follow-up, adherence to BSE over time, participation in screening programs, and clinical outcomes were not assessed. Because the intervention was delivered as an integrated educational program, the specific contribution of the wearable simulator could not be distinguished from those of the other educational components. The integrated educational intervention required one-on-one instruction and substantial personnel time, which may limit its scalability in community settings with limited resources. The analyses were based on complete cases rather than a strict intention-to-treat population. Five randomized participants with missing follow-up data were excluded without imputation, and attrition was unequal between the groups (one intervention and four control participants), which may have introduced attrition bias. Finally, the trial was registered retrospectively after the primary completion date. Consequently, the registry cannot establish that the study outcomes and statistical analyses were prespecified before the study data were examined, and this should be considered when interpreting the findings.

## Figures and Tables

**Figure 1:**
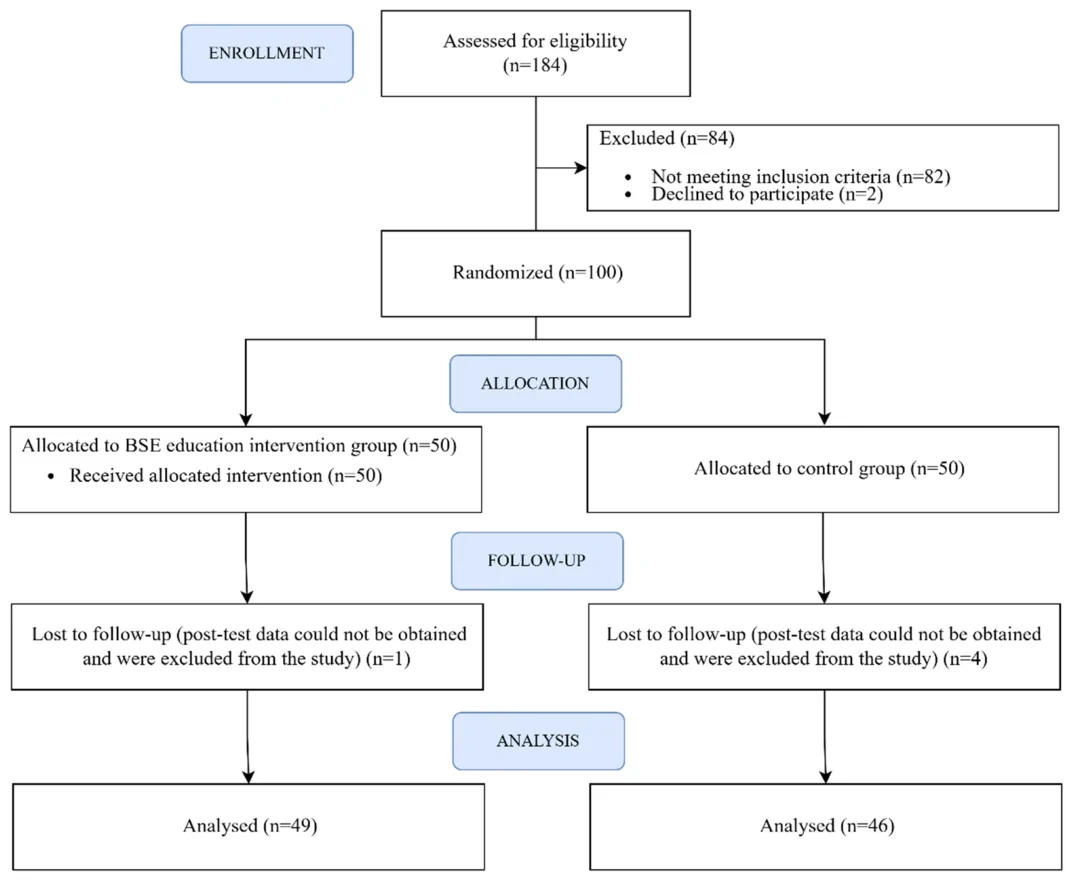
Consort Flow Diagram.

**Figure 2:**
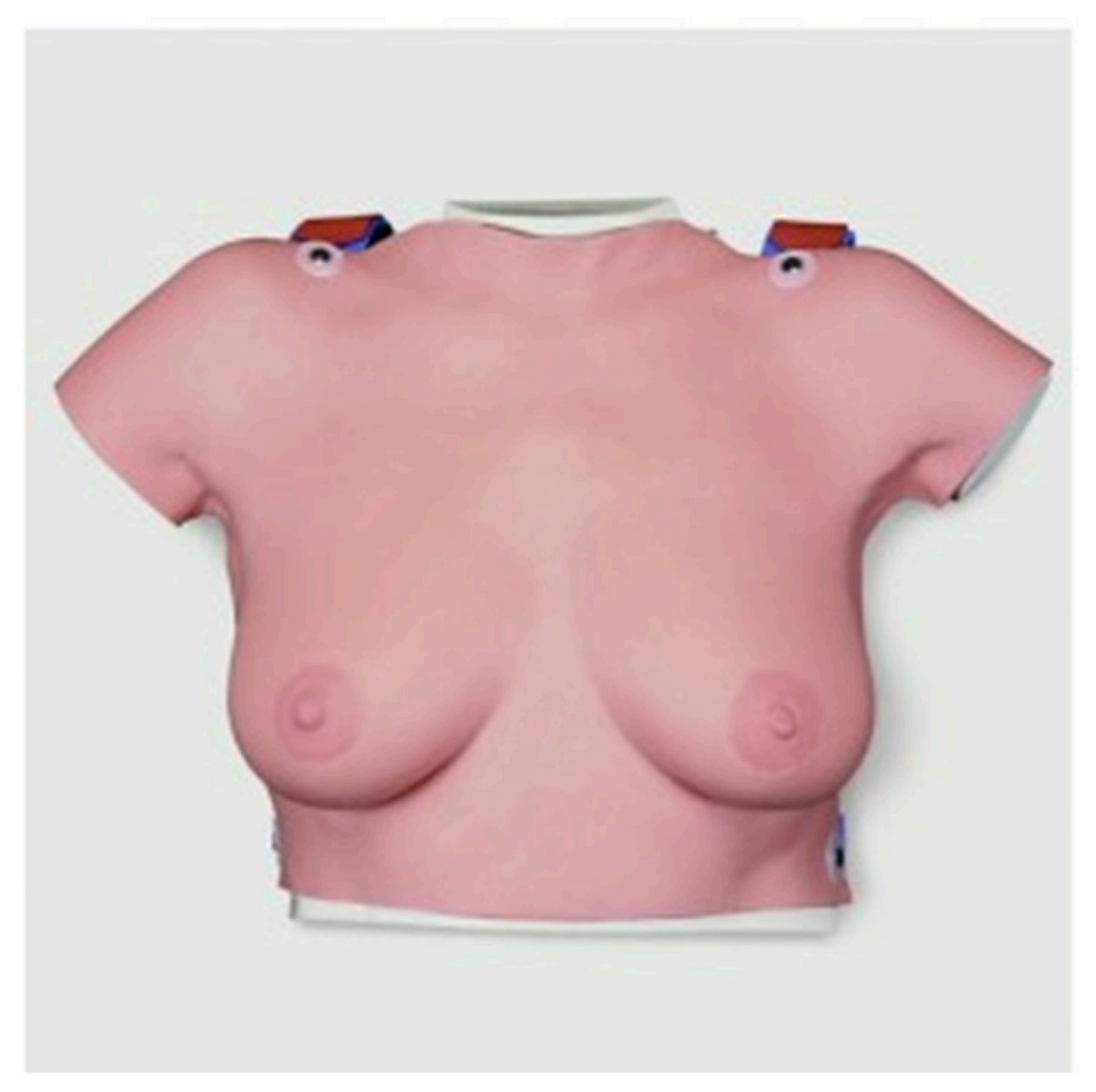
Wearable breast examination simulator (model L50) used during practical BSE training.

**Table 1:** Baseline characteristics of participants by study group.

Variables		Intervention (*n* = 49)		Control (*n* = 46)	
		*n*	%	*n*	%
Age (Years)	Mean ± SD	46.92 ± 6.39		48.59 ± 7.09	
	≤47	31	63.3	23	50.0
	>47	18	36.7	23	50.0
Marital status	Married	47	95.9	43	93.5
	Single	2	4.1	3	6.5
Education status	Primary School	24	49.0	33	71.7
	High school	16	32.7	10	21.7
	University and above	9	18.3	3	6.6
Family history of breast cancer	Yes	18	36.7	12	26.1
	No	31	63.3	34	73.9
If yes, the person with cancer (*n* = 18/12)	Mother	1	5.6	3	25.0
	Sibling	2	11.1	3	25.0
	Other	15	83.3	6	50.0
Family history of other cancers	Yes	26	53.1	21	45.7
	No	23	46.9	25	54.3
Number of births	Mean ± SD	3.00 ± 1.02		3.20 ± 1.31	
	≤2	14	28.6	9	19.6
	3	21	42.9	20	43.5
	≥4	14	28.6	17	37.0
Previous breastfeeding experience	Yes	48	98.0	43	93.5
	No	1	2.0	3	6.5
Total duration of breastfeeding	≤12 months	15	31.2	12	27.9
	>12 months	33	68.8	31	72.1

Data are presented as mean ± standard deviation or *n* (%).

**Table 2:** Descriptive Pre-Test and Post-Test Breast Cancer Knowledge Scores by Study Group.

Variables	Groups	Pre-Test	Post-Test
		Mean ± SD	Mean ± SD
General Knowledge	Intervention	5.60 ± 1.84	7.65 ± 1.73
	Control	6.22 ± 1.87	5.89 ± 1.52
Curability	Intervention	4.73 ± 2.08	5.48 ± 1.54
	Control	4.46 ± 1.62	4.17 ± 1.23
Comprehensive Breast Cancer Knowledge Test Total Score	Intervention	10.33 ±3.07	13.13 ± 2.69
	Control	10.67 ±2.79	10.07 ± 2.05

Data are presented as mean ± standard deviation (SD).

**Table 3:** Comparison of Baseline-Adjusted Post-Test Breast Cancer Knowledge Test Scores Between the Groups.

Variables	Intervention Group Adjusted Mean (95% CI)	Control Group Adjusted Mean (95% CI)	Adjusted Mean Difference (95% CI)	F(1, 92)	*p*	Partial η^2^
General Knowledge	7.71 (7.23–8.18)	5.90 (5.41–6.39)	1.81 (1.12–2.50)	27.05	<0.001	0.227
Curability	5.54 (5.13–5.94)	4.17 (3.75–4.58)	1.37 (0.79–1.95)	22.05	<0.001	0.193
BCKT Total Score (Sensitivity Analysis without Interaction)	13.23 (12.53–13.93)	10.08 (9.36–10.81)	3.14 (2.14–4.15)	38.44	<0.001	0.295
Exploratory Conditional Comparisons for the BCKT Total Score						
**Baseline BCKT Total Score Level**	**Intervention Group** **Adjusted Mean**	**Control Group** **Adjusted Mean**	**Adjusted Mean Difference (95% CI)**		***p***	
Low (−1 SD; 7.51)	13.05	10.97	2.08 (0.67–3.50)		0.004	
Mean (10.45)	13.26	10.13	3.13 (2.14–4.12)		<0.001	
High (+1 SD; 13.40)	13.46	9.29	4.18 (2.78–5.58)		<0.001	

Note. Positive adjusted mean differences favor the intervention group. Adjusted means and group comparisons were obtained using analysis of covariance (ANCOVA).

**Table 4:** Descriptive Pre-Test and Post-Test Champion Health Belief Model Scale Subscale Scores by Study Group.

Variable	Group	Pre-Test	Post-Test
		Mean ± SD	Mean ± SD
Susceptibility	Intervention	7.23 ± 3.07	8.31 ± 2.71
	Control	7.76 ± 3.99	9.00 ± 3.01
Seriousness	Intervention	19.60 ± 5.57	23.25 ± 5.13
	Control	21.04 ± 5.78	23.15 ± 5.06
Benefits	Intervention	19.67 ± 4.29	19.83 ± 3.21
	Control	19.17 ± 4.10	17.28 ± 3.26
Barriers	Intervention	25.06 ± 5.87	30.00 ± 6.58
	Control	31.61 ± 8.65	31.87 ± 6.86
Confidence	Intervention	27.54 ± 10.71	37.50 ± 6.61
	Control	33.72 ± 9.35	31.13 ± 6.99
Health Motivation	Intervention	24.71 ± 5.30	25.83 ± 4.67
	Control	25.91 ± 4.80	21.85 ± 4.86

Data are presented as mean ± standard deviation (SD).

**Table 5:** ANCOVA Results for CHBMS Subscales.

CHBMS Subscale	Intervention Adjusted Mean (95% CI)	Control Adjusted Mean (95% CI)	Adjusted Mean Difference (95% CI)	F(1, 92)	*p*	Partial η^2^
Susceptibility	8.33 (7.56–9.11)	8.95 (8.15–9.75)	−0.62 (−1.73–0.50)	1.21	0.274	0.013
Seriousness	23.36 (21.90–24.82)	23.14 (21.63–24.64)	0.22 (−1.88–2.33)	0.05	0.833	<0.001
Benefits	19.81 (18.89–20.73)	17.29 (16.34–18.23)	2.53 (1.21–3.85)	14.42	<0.001	0.136
Barriers	30.32 (28.33–32.31)	31.53 (29.47–33.59)	−1.20 (−4.20–1.79)	0.64	0.427	0.007
Confidence	37.60 (35.62–39.57)	31.10 (29.06–33.15)	6.49 (3.59–9.40)	19.66	<0.001	0.176
Health motivation	25.91 (24.55–27.28)	21.83 (20.43–23.24)	4.08 (2.12–6.05)	17.00	<0.001	0.156

Note. Adjusted means were estimated using baseline scores as covariates. Mean differences are intervention minus control. Assumptions of homogeneous regression slopes and error variances were met for all subscales (all *p* > 0.05). The Bonferroni-adjusted significance threshold was *p* < 0.008. CI, confidence interval; CHBMS, Champion’s Health Belief Model Scale.

## Data Availability

The data supporting the findings of this study are available from the corresponding author upon reasonable request. The data are restricted based on participant privacy, confidentiality, and the conditions of informed consent.

## Supplementary Materials

The following supporting information can be downloaded at: https://www.mdpi.com/article/10.3390/healthcare14193191/s1, Table S1: Comparison of breast cancer knowledge scores between and within the study groups; Table S2: Comparison of pre-test and post-test Champion Health Belief Model Scale subscale scores between the intervention and control groups.
