# Supplementary material for: Effects of an Integrated Breast Self-Examination Education Program Incorporating Practice with a Wearable Breast Examination Simulator on Breast Cancer Knowledge and Health Beliefs: A Randomized Controlled Trial
Source: Healthcare (Basel). 2026 Sep 28;14(19):3191. doi: 10.3390/healthcare14193191 (PMC13665267; doi:10.3390/healthcare14193191)
Supplement: Supplementary file 1 [file healthcare-14-03191-s001.zip › healthcare-4532206-supplementary.pdf]

Supplementary Table S1. Comparison of breast cancer knowledge scores between and within the study groups

Variables	Groups	Pre-Test Mean ± SD	Post-Test Mean ± SD	t	p
General Knowledge	Intervention	5.60 ± 1.84	7.65 ± 1.73	-6.062***	**<0.001***
	Control	6.22 ± 1.87	5.89 ± 1.52	0.848***	0.401
	Test value	-1.600**	5.206**		
	p	0.113	**<0.001***		
Curability	Intervention	4.73 ± 2.08	5.48 ± 1.54	-2.053***	**0.046***
	Control	4.46 ± 1.62	4.17 ± 1.23	0.853***	0.398
	Test value	0.708**	4.515**		
	p	0.481	**<0.001**		
Comprehensive Breast Cancer Knowledge Test Total Score	Intervention	10.33 ±3.07	13.13 ± 2.69	-5.123***	**<0.001***
	Control	10.67 ±2.79	10.07 ± 2.05	1.031***	0.308
	Test value	-0.561**	6.178**		
	p	0.575	**<0.001***		

*p<0.05 **<0.01 ***<0.001; independent-samples t-test was used for between-group comparisons, and paired-samples t-test for within-group comparisons

At baseline, no significant between-group differences were observed in the BCKT General Knowledge, Curability, or total scores (all p>0.05). At post-test, all three scores were significantly higher in the intervention group than in the control group (all p<0.001). Within the intervention group, General Knowledge, Curability, and total scores increased significantly from pre-test to post-test (all p<0.05), whereas no significant changes were observed in the control group (all p>0.05) (Table 2).

Supplementary Table S2. Comparison of pre-test and post-test Champion Health Belief Model Scale subscale scores between the intervention and control groups

Variable	Group	Pre-Test Mean ± SD	Post-Test Mean ± SD	Within-group t	p
Susceptibility	Intervention	7.23 ± 3.07	8.31 ± 2.71	-2.152	0.037*
	Control	7.76 ± 3.99	9.00 ± 3.01	-2.103	0.041*
	Between-group t	-0.726	-1.164		
	p	0.470	0.248		
Seriousness	Intervention	19.60 ± 5.57	23.25 ± 5.13	-3.380	0.001*
	Control	21.04 ± 5.78	23.15 ± 5.06	-1.873	0.068
	Between-group t	-1.230	0.093		
	p	0.222	0.926		
Benefits	Intervention	19.67 ± 4.29	19.83 ± 3.21	-0.209	0.836
	Control	19.17 ± 4.10	17.28 ± 3.26	2.585	0.013*
	Between-group t	0.569	3.819		
	p	0.571	<0.001*		
Barriers	Intervention	25.06 ± 5.87	30.00 ± 6.58	-3.827	<0.001*
	Control	31.61 ± 8.65	31.87 ± 6.86	-0.179	0.859
	Between-group t	-4.309	-1.350		
	p	<0.001*	0.180		
Confidence	Intervention	27.54 ± 10.71	37.50 ± 6.61	-5.239	<0.001*

	Control	33.72 ± 9.35	31.13 ± 6.99	1.620	0.112
	Between-group t	-2.973	4.540		
	p	0.004*	<0.001*		
Health Motivation	Intervention	24.71 ± 5.30	25.83 ± 4.67	-1.070	0.290
	Control	25.91 ± 4.80	21.85 ± 4.86	4.393	<0.001*
	Between-group t	-1.153	4.054		
	p	0.252	<0.001*		

*p<0.05. Between-group comparisons were performed using the independent-samples t-test; within-group pre–post comparisons were performed using the paired-samples t-test.

At baseline, the intervention group had significantly lower perceived barriers and confidence scores than the control group ($p<0.001$ and $p=0.004$, respectively), whereas no significant differences were observed in the other CHBMS subscales. At post-test, the intervention group had significantly higher perceived benefits, confidence, and health motivation scores than the control group (all $p<0.001$). Within the intervention group, susceptibility, seriousness, barriers, and confidence scores increased significantly from pre-test to post-test. Within the control group, susceptibility increased, whereas perceived benefits and health motivation decreased significantly (Table 4).
